# Lesbians’ attitudes and practices of cervical cancer screening: a qualitative study

**DOI:** 10.1186/s12905-014-0153-2

**Published:** 2014-12-12

**Authors:** Claire Curmi, Kath Peters, Yenna Salamonson

**Affiliations:** School of Nursing & Midwifery, University of Western Sydney, Locked Bag 1797, Penrith, NSW 2751 Australia

**Keywords:** Lesbian, Cervical cancer screening, Pap test, Pap smear, Papanicolaou test, Cancer prevention, Human papilloma virus

## Abstract

**Background:**

Cervical cancer is the third most prevalent cancer in women, and since the introduction of the Papanicolaou test (Pap test or Pap smear), the incidence of cervical cancer and mortality rates worldwide have declined substantially. However significant disparities have been identified between the cervical screening rates of heterosexual and lesbian women. This study explores the attitudes and practices that lesbians have towards cervical cancer screening and aims to identify why such disparities occur.

**Methods:**

A qualitative methodology based on feminist perspectives was used to collect narrative data from lesbians about their attitudes and practices of cervical screening through the use of semi structured interviews. Nine women who self-identified as lesbian that were living in New South Wales were recruited for the study. Interviews were digitally recorded and transcribed verbatim. Data were analysed using a thematic analysis approach.

**Results:**

Four main themes emerged from the data namely: Encountering cervical cancer: “my friends had some early cancer cells detected”, Misconceptions related to risk: “I am a lesbian I don’t need one”, Imposed screening: “It’s a requirement of IVF treatment” and, Promoting cervical screening: “I think it should be spoken about in schools”.

**Conclusions:**

Consistent with the literature, the findings show that the majority of these women do not undertake cervical screening at the recommended rate. This study highlights the multiple and complex issues related to cervical cancer screening for lesbians, mainly through misconceptions and underestimation of risk. Specific and targeted educational and promotional strategies are required for both lesbians and health professionals to enhance cervical cancer screening rates for lesbians in Australia.

## Background

The leading cause of death in developed countries is cancer [1]. After breast and colorectal cancer, cervical cancer is considered to be the third most prevalent cancer in women [1,2]. In fact, one in ten diagnosed cancers in women are that of the cervix [3] resulting in 275,000 deaths worldwide in 2008 [2,4]. Cervical cancer is often diagnosed in young adult women whom are often primary carers for their families or actively involved in their careers [2]. Women who survive cervical cancer often have ongoing physical problems such as bladder and bowel dysfunction and painful sexual intercourse, which can have a calamitous effect on their quality of life [5].

The Papanicolaou test (Pap test or Pap smear) is an effective and important screening tool used worldwide to detect pre-cancerous cervical abnormalities that can be treated early to prevent the progression to invasive carcinoma [6-8]. Since the introduction of regular screening with the Pap test, the incidence of cervical cancer and mortality rates worldwide have declined substantially [6]. This decline has also occurred in Australia, however, in 2009 there were 631 new cases diagnosed and in 2010, 152 deaths resulting from cervical cancer [9].

In Australia, the goal of the National Cervical Screening Program is to decrease morbidity and deaths from cervical cancer through a systematic approach to cervical screening, aimed at identifying and treating irregularities before potentially developing into cervical cancer [9]. Australia’s national policy for screening recommends that “all women who have ever been sexually active should start having Pap smears between the ages of 18–20, or one to two years after having sexual intercourse, whichever is later” [10 p. 5]. The cervical cytology program is crucial to Australia’s cervical screening program as it provides reminder letters to women who are overdue for screening, collaborates with health care professionals to ensure women who have had an abnormal result have received adequate follow up care, as well as providing a registry with data and history for the national monitoring of the program [10]. However, this program is only effective at reaching women who have participated in cervical cancer screening.

Pap smear screening rates have been shown to vary between different socio-demographic groups. Studies have shown that women who are less educated, have a low socioeconomic status, live in rural areas or are older, tend to have lower rates of cervical screening [6,7]. Further, subpopulations have been identified as having lower cervical screening rates than the general population, such as those from racial/ethnic minority groups [11], women with developmental or physical disabilities [12-15], and women who identify as being lesbian [6,11,16].

Previous research has fuelled a common belief that sexually transmitted infections (STIs) are scarcely transmitted between lesbians [17-19]. This has contributed to the limited research that focusses on the health risks encountered by them, including their practices of cervical cancer screening [20,21]. However recent data strongly supports that STIs are common in lesbians with substantial evidence that transmission occurs through lesbian sexual contact [18]. Despite these recent findings, lesbians and health professionals alike reportedly lack awareness of the possibility of STI transmission during woman to woman sexual contact.

The lack of knowledge regarding mode of transmission of STIs by both lesbians and health care professionals has been identified as a significant barrier to cervical screening [22]. A contributing factor to the lack of awareness could be that the sexual practices of lesbians have been compared to that of virgins or nuns [23], whilst research conducted by Barnes found as many as 30% of lesbians studied were unaware that HPV could be spread from woman to woman during sexual activity [24]. As human papillomavirus (HPV) is considered to be transmitted predominately through heterosexual penetrative intercourse [25], lesbians are often thought to be at lower risk of cervical cancer than heterosexual women [26]. Therefore many health care professionals remain unaware of the importance of cervical screening in this group [27,28]. However this is not the case as shown in Grindel, et al’s study of 1139 lesbians, 79.6% of participants reported having an abnormal pap smear, with several other studies documenting cases of cervical cancer in lesbians who have never had heterosexual sex [17,23,29,30].

While several studies have identified significant disparities between the cervical screening rates of heterosexual and lesbian women [6,31-33], further research is required to identify why such disparities occur. The aim of this paper is to highlight attitudes and practices of lesbians related to cervical cancer screening.

## Methods

### Design

A qualitative methodology based on feminist perspectives was used to collect narrative data from lesbians about their attitudes and practices of cervical screening. As the central concern of feminist research is to identify and understand the fundamental values of women’s ways of thinking, doing and being [34], a qualitative methodology based on feminist principles was deemed appropriate for this study as feminist research aims to study and explain the social world through the views and experiences of women [35].

### Recruitment

Prior to recruitment approval was gained from the University of Western Sydney Human Research Ethics Committee. Women aged between 18 and 69, living in New South Wales, who self-identified as lesbians were recruited via media release and social media and invited to participate in an interview. The use of social media allowed the message to be shared amongst others in their social circle for widespread dissemination [36]. Once initial contact was made, snowball sampling was used to recruit further participants by asking if they knew of any other people fitting the inclusion criteria. They were then asked to forward the details of the study, including the researcher’s contact details to potential participants. As lesbians are a minority and potentially marginalised [6,37] this sampling method overcame the often challenging task of recruiting difficult-to-find participants [36].

### Data collection

In-depth interviews were conducted on a one-to-one basis with the women choosing to be interviewed either face-to-face, or via telephone. The interview duration was approximately one hour and face-to-face interviews were held at either a university campus or at the participant’s place of residence. The aim of data collection was to explore in-depth, the attitudes and practices that lesbians have towards cervical screening. Therefore, an interview guide was used and the questions asked of the women during the interviews were open-ended, which facilitated the dialogue-sharing process [38]. Congruent with feminist perspectives, further strategies were utilised to build rapport to minimise any power imbalances between the participants and researcher [39]. This was achieved by investing time with participants and making sure they were comfortable during the interview process. Both face-to-face and telephone interviews were recorded on a digital recorder and later transcribed. This transcribed data along with the online interviews formed the textual data for analysis.

### Data analysis

On completion of each interview, all participants’ narratives were transcribed verbatim by the researcher. This was completed immediately following each interview to ensure that the conversations were documented as precisely as possible. The completed transcripts were then read and re-read to ensure the researcher immersed themselves in the data. By reading and re-reading the data it allowed the researcher to detect patterns and commonalities across the data collected [40]. Similar sentences, phrases or words that denoted a similar notion were grouped together to form a category. The sections encompassing each category were amalgamated and summarized to form general themes that largely represented the women’s attitudes and practices related to cervical screening [41].

### Ethical considerations

Ethical approval was gained from the Institutional Ethical Review Board prior to recruitment of participants. Before being interviewed, participants were asked to sign a consent form and, as the topic of cervical screening is sensitive in nature, participants were also provided with a list of counselling services for them to use if required.

As it was not known whether participants publicly identified themselves as lesbian, disclosure of this may have had potentially negative implications for participants. Therefore provisions were made to ensure their privacy and confidentiality. Their decision to participate in the research was kept confidential and interviews were conducted in a private space, outside the audible range of others. To further protect their privacy and maintain confidentiality, the researcher de-identified the interviews during transcription, using pseudonyms to protect participants’ identities [42]. Furthermore, any locations or people named by participants during the interview were changed and only general, non-identifiable terms were used in the interview transcripts and in the findings presented. To ensure participants are not identifiable by their stories, qualitative findings are presented collectively rather than individually [42].

### Rigour

For the purpose of this study, rigour was achieved through the use of reflexivity, and by ensuring credibility and auditability. Reflexivity is the act of the researcher outlaying personal beliefs, preconceptions and one’s own biases [43]. Reflexivity was achieved was by participating in self-reflection which gave the researcher the ability to recognise the influence that her beliefs and values may have had on the type and quality of the data collected [44,45]. In order to ensure the practice of self-reflection, the researcher also kept a reflective journal which was updated after each interview.

In order to achieve credibility of the study, the researcher personally transcribed all of the interviews and listened to audio recording whilst reading transcripts. This not only gave the researcher an in depth sense of the data, it also aided in the development of skills required in both the reflection and transcription process. Furthermore it assisted in increasing the accuracy of both content and context of the data collected, thus ensuring credibility [46]. Auditability is the ability of another investigator to follow the decision trail in order to determine the degree to which consistency has been achieved in the study [47,48]. In the current study, auditability was achieved by co-investigators checking transcripts and findings ensuring they were able to follow decisions made by the researcher in the interpretation of the data.

## Results

In total, nine women who self-identified as lesbians volunteered to participate in the study. Of these, two had never been screened for cervical cancer, four had been screened once, and three were screened on a regular basis (Table 1). The data analysis of the qualitative data resulted in four themes that encapsulated the attitudes and practices of lesbians related to cervical cancer screening. The first theme, ‘Encountering cervical cancer’ highlights that although some participants had friends or family that had been diagnosed with cancer, this experience had mixed effects on their own screening practices. The second theme, ‘Misconceptions related to risk’ highlighted misunderstandings held by participants, their friends and family and some health professionals about the necessity for lesbians to undergo cervical screening. The third theme ’Imposed screening’, highlights that those participants trying to have children, especially through in vitro fertilisation (IVF), have required by their clinics or doctors to undertake a cervical screen before treatment. All of the participants who had undertaken cervical screening as a requirement of receiving assisted reproductive technology (ART) stated they would have not done so otherwise. The final theme, “Promoting cervical screening”, highlights participants’ reflections after participating in the study and their acknowledgement of the need for more education and health promotion related to cervical cancer screening.

**Table 1:** **Participant demographics**

	**Age category**	**Country of birth**	**Employment status**	**History of sex with a male**	**Cervical screening history**	**Current relationship status**	**Plans for (more) biological children**
**Sophie**	Early 20s	Australia	Health-related work	Yes	Once only, intent to screen regularly	Living with a partner who has children from previous heterosexual relationship	No
**Olivia**	Mid-20s	Australia	Health-related work	Yes	Once only several years ago	Living with a partner	No
**Kayla**	Mid-20s	Australia	Non-health-related work	Yes	Regular screening	Living with a partner	No
**Anna**	Mid-20s	Overseas	Health-related work	Yes	Regular screening	Living with a partner	No
**Amelia**	Mid-30s	Overseas	Not in paid work, a student	Yes	Once only, no intention for regular screening	Living with a partner, with two children	Yes
**Grace**	Mid-30s	Australia	Not in paid work, 4 children	No	Regular screening	Living with a partner, with children	Yes
**Charlotte**	Mid-30s	Australia	Non-health-related work	No	Never and no intention to do so	Living with a partner with 2 non-biological children	Yes
**Molly**	Late 30s	Australia	Non-health-related work	No	Once only, intent to screen regularly	Living with a partner	No
**Skye**	Early 40s	Australia	Health-related work	No	Never and no intention to do so	Living with a partner	Yes, partner undergoing fertility treatment

### Encountering cervical cancer “*My friends had some early cancer cells detected*”

Most of the women who participated in this study had encountered cervical cancer at some stage of their lives. These encounters were through the diagnoses of partners, friends or family members. Some of the women who participated in this study had friends or partners that had cervical cancer detected early, and this motivated them to initiate screening. Sophie and Grace conveyed this sentiment in the following quotes.

*One of my ex partners had trouble in the past with pre-cancerous cells. She had an irregular pap smear before I was with her sexually but she had a pap smear while we were together and it was irregular, so I thought it was quite important that I go and check, thankfully it was all fine. So that really made me want to go, but I was a bit ignorant prior to that. (Sophie)*

*Two of my friends had some early cancer cells picked up in theirs so it’s just kind of like you know something you do just…to be sure (Grace)*

However, despite having a family history of cervical cancer some participants chose not to be screened or have regular preventative screening. Charlotte conveys:

*I understand my mother has had cervical cancer, my sisters have, I understand all of that it runs in the family you know (Charlotte)*

Despite this Charlotte continues not to screen and when asked why, she explains that she did not consider herself at risk of cervical cancer as relates such risk with sexual relations with a male and promiscuity.

*‘cause my grandmothers were fine, my mother was a bit of a tramp, my sisters a tramp and so I think multiple partners and multiple I don’t know it could be something to do with that you know and I’m not like that and I haven’t been with a guy or had multiple partners (Charlotte)*

Olivia, who has only ever been screened once, also had family members with a history of abnormal cervical screening results. Despite this knowledge, the following excerpt indicates her perception that she is not at risk, seemingly conveying her belief that a biological link is a greater indicator of risk than sexual contact.

*When I first got one done [cervical screen] I pretty much had my mother in my ear telling me that we all need to get pap smears cause I have two sisters and it’s been like her mantra that we must have a pap smear because she has had she had a bit of a scare when she was younger…we are not her biological [children] (laughs) so you know I am safe (laughs) …actually her biological daughter did have the same thing, it’s like the HPV virus but they burn out (Olivia)*

### Misconceptions related to risk: “*I am a lesbian I don’t need one*”

There was some incongruity between what the women vocalised and their screening practices. This was due to multiple factors, including confusion around sexual practices and their perceived risk of being diagnosed with cervical cancer due to the transmission of HPV. Adding to participants’ confusion was the Australian national policy for cervical cancer screening. These guidelines advise women to commence cervical screening at 18 years of age or two years after becoming sexually active, whichever comes first. This raised questions from participants regarding what constituted ‘sexual activity’ in this context. Olivia who has only undertaken taken cervical screening once explained:

*I think that’s where it comes into play as well this whole idea that and I have had this question all throughout my life of where but you haven’t really had sex or have you actually had sex though?....and that’s what I think too and maybe that’s something that kind of contributes to that because lesbian sex isn’t considered…because it’s not penetrative you know man, woman, sex (Olivia)*

A common belief amongst participants was the misconception that HPV was transmitted only via men. They perceived that because they were lesbians and not having sexual intercourse with men, they were not at risk of contracting HPV and were subsequently not required to undertake cervical screening. The following excerpts illuminate this perception shared by Molly and Skye.

*Yeah well I was under the impression that because I had never had sex with a bloke that I didn’t need to have one [Pap smear] (Molly)*

*I think lesbian women who have been previously sexually active with a male who may have come in contact the HPV virus, which is what my understanding one of causes cervical cancer definitely would be important, however for lesbians who may have never had any sexual contact with male sexual intercourse I don’t think it’s important (Skye)*

The belief held by these women that men’s body fluids were the main vectors of HPV extended to all STIs with the majority of participants believing that the spread of STIs from woman to woman was not likely.

*I think if you have unprotected sex with a guy then there is a higher risk of contracting something than if you have a night out with a girl and take her home, because really there is no, well in my experience there is not that much contact between you know a little bit obviously (laughs) let’s not get that into it but you know there’s not so much interaction between those parts that would transmit I suppose that (Olivia)*

*Most lesbians don’t touch parts for there to be that transfer [of body fluids] so if you were using your hands you would wash them and if you were using implements you would wash them so I would never use the same implement as someone else with stuff on it so our juices would never touch in way and her juices would not touch my private parts if that makes sense (Molly)*

Due to participants’ perceptions that their risk of contracting a STI was reduced, they reported not practising safe sex, which places them at a higher risk of contracting HPV. This is illustrated in the following quote by Olivia.

*There is not this big thing on STDs (sexually transmitted disease) [for lesbians] that there seems to be on more heterosexual communities…like I know that it doesn’t stop you from getting STDs and stuff from being with women but I am guilty of doing the whole…like not assuming that a female could actually pass on something to me. I don’t know maybe it’s not stressed as much in our circles that you need to use protection because I would be offended if someone took like you know those dams [dental dam] and stuff I would be pretty offended if someone took one of those out (Olivia)*

Of additional concern was the fact that the misconception held by participants that lesbians were not at risk of cervical cancer, and therefore did not require preventative screening, often stemmed from advice offered by their health care providers. Participants reported being told by health professionals that they did not need to undertake cervical screening because they had not had sexual intercourse with men. This information was readily accepted by these women and as a consequence, some participants had never had a Pap test. Amelia, has only ever untaken one cervical screen after the birth of her first child. Here she discusses the information she received from a doctor advising her that because she was a lesbian she did not require cervical screening.

*I have had doctors think that it’s not even necessary for lesbians like the first doctor I ever brought it up to, I must have been 18 and he was like oh no you don’t need one because I didn’t have sex with a man and I yeah so you know and at 18 I was pretty happy to take that explanation (laughs) and run away, yeah it was good enough for me (laughs) (Amelia)*

Skye, who had never been screened nor had any intention to be screened talks about receiving information on not requiring screening as a lesbian.

*I did some research and spoke to a few Doctors because I was working in the department of medicine and I told them that I had never been sexually active with a male and they said that I didn’t need to have a pap test, which was probably twenty odd years ago. (Skye)*

Misunderstanding regarding the necessity for cervical cancer screening also extended to family and friends. Many of the women who participated in this study also relied on their mothers, sisters and female friends for advice on whether they needed to screen or not.

*So it might have been a discussion between my mum and sister about pap smears and I’m like ‘ha sucked in you guys have still got to get pap smears’ and they were like’ yeah you lucky bitch you don’t have to’ (Molly)*

### Imposed screening: “*It’s a requirement of IVF treatment*”

Some participants indicated that the only reason they had undertaken cervical cancer screening was to comply with pre-treatment requirements for assisted reproductive technology (ART) or routine screening during and after pregnancy. None of the participants who were undertaking or had undertaken ART had been screened prior to commencing treatment.

*[I have a Pap test] Generally every two years but it’s a requirement of IVF treatment that you have one before you commence treatment, so whether you have had one 6 months ago, 12 months ago or whatever the fertility clinic that we use it’s a requirement that you have one within sort of the last two to three months before you commence treatment. (Grace)*

*They [doctors] were fairly forceful about getting it [Pap smear] after pregnancy they really pushed and I did it. (Amelia)*

Participants also mentioned they had a number of friends who were undergoing ART who had previously never undertaken cervical screening and would have not done so if it was not for the requirement of the IVF clinic.

*It’s a case with some of my friends that I do know that until they started the process of IVF they hadn’t had one [cervical screen] you know one of my friends oh she was 32 when she went first through IVF and she had never had one [cervical screen] (Grace)*

Skye, who had never been screened nor had any real intention to screen, talked about pregnancy being the only reason she would undertake cervical screening.

*…if I did need to have one there would only be one reason I would undertake a pap smear test and that was if I was going to try and get pregnant. (Skye)*

### Promoting cervical screening: “*I think it should be spoken about in schools*”

This study clearly provided the participants with the opportunity to reflect on their own screening knowledge, and how this related to their own screening behaviours and beliefs. As a consequence, all participants agreed that there is a need for more lesbian specific information in the public arena about cervical screening. Amelia conveys:

*You see a lot of awareness campaigns for getting Pap smears but I don’t think I have ever seen one directed at lesbians (Amelia)*

Participants also made suggestions for greater content regarding lesbians to be included in sex education classes taught in high school. They perceived that this was necessary to ensure a clear message was received about the importance of cervical cancer screening for all women, not just those who were heterosexually inclined.

*[At school] you actually get taught sexual health as a straight person [so] when you have finally and are comfortable to come out you think that that sexual education doesn’t necessarily apply to you because of your sexual orientation. So I think it should be actually be spoken about in schools (Kayla)*

The women acknowledged that despite health concerns for gay men being widely publicised, health concerns for lesbians were not. Participants discussed the potential for the media to highlight the importance of cervical cancer screening for lesbians.

*I think obviously addressing the importance of it in in various publications like Lesbians on the loose (LOTL) and things like that that are out there for you know specifically designed for lesbians and putting it out there and cause a lot of lesbian friendly doctors advertise in that and stuff and nurses and clinics advertise in that so yeah making those people making it known as well that how important it is (Grace)*

## Discussion

### Underestimation of risk

Overall, participants of the current study perceived that lesbians had little to no risk of cervical cancer. Many of the women conveyed the perception that they did not need to undertake a Pap test as they were lesbians and not engaging in sexual intercourse with men. Similar findings are evident in previous research by Fish [26] who found the most prevalent answer for perceptions of lesbians being at lower risk of cervical cancer were that of no sexual intercourse with men.

Fish and Anthony [49] conducted a survey of the health behaviour of 1066 lesbians in the UK. 50% of lesbians surveyed perceived their risk of cervical cancer to be less than that of heterosexual women. Among these respondents, women who perceived themselves as having no risk were more likely to have never attended screening. The belief of being at more risk due to heterosexual intercourse was also evident in the current study, as participants who screened regularly had at some point in their lifetime engaged in sexual intercourse with a male. This factor was highlighted by all participants as a reason as to why or why not to participate in cervical screening. The perception of being at less risk can have a detrimental effect on their health as several studies have documented cases of cervical cancer in lesbians who have never had heterosexual sex [17,23,29].

Two women in the current study who had never been screened, nor had any intention of being screened, had never engaged in sexual intercourse with a male. Similarly a study conducted by Marrazzo, et al. [50], found the screening practices among lesbians to be closely linked to previous or current sex with men and those that were exclusively lesbian were significantly different from lesbians who had experienced heterosexual intercourse, in that their first cervical screen had occurred at an older age, and they have fewer screens with longer intervals between screens.

Marrazzo, et al’s [50] study also showed that 95% of lesbian participants believed they should undertake cervical screening at the recommended intervals. However, despite this acknowledgement, confusion is clearly evident in terms of their perceived risk as 36% of these participants cited the most common reason for not undertaking a Pap test within the previous two years was their belief of not needing a cervical screen because they were not engaging in sexual intercourse with men [50]. This is consistent with previous research, with participants of the study of having no or little risk of cervical cancer [17,23,29]. It is imperative that information given to lesbians regarding screening is accurate as, even though lesbians are thought to be at lower risk of cervical cancer, several studies have found cervical abnormalities women who are exclusively lesbian [18,22,51]. This information highlights the importance that all women, regardless of sexual behaviour, undertake regular cervical screening.

Many of the participants had received incorrect advice about screening practices. The lack of knowledge related to the cervical screening needs of this group of women was not limited to themselves, but also their friends and health professionals. Studies have shown that a woman’s social network, particularly that of female friends and family have the strongest influence on the use of preventative health behaviours such as cervical screening [52]. Whilst most of the participants knew someone that had had an abnormal pap smear, this fact had encouraged two participants to undertake screening, however it had no effect on the screening practices of the majority of women in this study.

Several previous studies highlight issues with health care providers advising lesbians that they do not require cervical screening [22,28,53], with many women believing and relaying this inaccurate message [22,53]. This incorrect dissemination of information hinders screening practices and this is shown in the study with two of the participants receiving incorrect advice on screening in lesbians and as such chose not to screen.

### Misconceptions: STIs/HPV and safe sex

There is a common belief that STIs are rarely transmitted between women [20,21]. This belief regarding the transmission of STIs, specifically HPV transmission was also evident in the study with only one participant identifying the possibility of HPV transmission between women. Interestingly this participant had never been screened nor had any intention of being screened. However the other participants believed they were not at risk of contracting HPV or any other STI as they felt their sexual practices did not facilitate the transmission of such infections. Whilst they understood how STIs are transmitted via penetrative heterosexual sex, they found it difficult to comprehend how STIs could be transmitted between women. This uncertainty about what constituted safe sex practices for lesbians was a major contributor to the absence of safe sex practice amongst all participants.

Several previous studies have reinforced the lack of knowledge lesbians have towards HPV transmission between women with as many as 50-94% of lesbians not knowing or believing that HPV could be spread from one women to another during sex [21,54]. Whilst it was evident in the study that the participants were unaware of the safe sex needs of lesbians, a study conducted by McNair [54], reports that 18.6% of women with a lifetime of female partners reported at least one abnormal cervical screen highlighting the importance of safe sex practice in this group of women.

It is believed that because lesbians do not have sexual contact with men they are unlikely to carry the HPV and therefore do not need to undertake cervical screening [20,51,55]. This was apparent in the current study with the women revealing they considered semen to be the carrier of HPV. Therefore they perceived that if they were not currently having intercourse with a male, they were not at risk of developing cervical cancer, thus negating the need for regular cervical screening. However, several studies have identified HPV to be prevalent amongst lesbians, with HPV DNA identified in 13%- 30% of lesbians [22,50] with 19%-21% of these women reporting in never having engaged in sexual intercourse with a male [22,56]. Whilst there is a common belief that lesbian women do not have sex with men [21,57], findings from this study revealed that four of the nine participants in this current small study had disclosed that they had engaged in sexual intercourse with a male, and this resonates with several studies that found large numbers of lesbians reported past or present sexual activity with men [17,58].

With recent publicity in Australia in regards to the HPV vaccine, a programme initiated by the Australian government commenced in 2007 to provide school aged children, (both male and female aged 12 to 15 years), with a vaccine against the most common strains of HPV [59]. Whilst it is predicted that the incidence of cervical cancer will decline substantially due to the vaccine, the effect of this vaccine is already being seen with an Australian study reporting a 77% decrease in HPV related infections [60]. Whilst these results are favourable, many women continue to choose not to participate in the vaccine program and as such places them at a greater risk of developing cervical cancer [61].

### Implications for research and practice

This study highlights the need for further research exploring health professionals’ awareness of the cervical screening needs of lesbians. Such research has the potential to illuminate misconceptions and inform recommendations for educational strategies. Additionally, further research on lesbian safe sex is required to enhance knowledge of the risk of HPV and promote safe sex in lesbian women. Further, education on the importance of cervical screening in lesbians needs to be addressed on a larger scale with the majority of participants suggesting that it should be included in school curricula and advertised in the gay media.

## Conclusions

This study aimed to gain information from lesbians to understand the attitudes and practices they have towards cervical screening. The findings show that the majority of these women do not undertake cervical screening at the recommended rate, which is consistent with current literature. The women themselves believe they are at less risk of cervical cancer, often reinforced by incorrect advice from health professionals, family and friends. The participants acknowledged there is a lack of safe sex being practiced and had a poor understanding of HPV, especially in regards to HPV being transmissible from woman to woman. All participants agreed that further promotion and education is required, on both the cervical screening needs specific to lesbians and the importance of the safe sex practices, to keep women safe and help prevent the transmission of STIs.
